# Bufei Jiedu Formula enhances CD40 activation and macrophage polarization to eliminate intracellular MRSA persisters

**DOI:** 10.3389/fimmu.2025.1623182

**Published:** 2025-07-17

**Authors:** Shaoyan Zhang, Jiajun Chen, Lei Qiu, Xianwei Wu, Wei Zhou, Ruoqing Peng, Ya Feng, Rui Zhou, Xing Huang, Dingzhong Wu, Zhenhui Lu

**Affiliations:** Institute of Respiratory Diseases, Longhua Hospital, Shanghai University of Traditional Chinese Medicine, Shanghai, China

**Keywords:** MRSA infection, persistent, intracellular, macrophage polarization, CD40, atractylenolide II

## Abstract

**Background:**

Intracellular methicillin-resistant *Staphylococcus aureus* (MRSA) represents a complex infection in clinical practice, characterized by its refractory and recurrent nature, rendering it challenging to treat with conventional antibiotics. Bufei Jiedu Formula (BFJD) is a traditional Chinese medicine compound utilized for treating chronic lung infections; however, its mechanisms against intracellular MRSA infection are not yet fully understood.

**Methods:**

An animal model with persistent MRSA infection was used to evaluate the efficacy of BFJD against chronic bacterial infections. Flow cytometry was employed to assess the regulatory effects of BFJD on macrophages. Transcriptomic sequencing and molecular biological experiments were utilized to explore and validate the regulatory targets and pathways of BFJD. Flow cytometry and molecular docking were used to clarify the possible binding mode of bioactive compounds with CD40.

**Results:**

BFJD reduced bacterial loads in the lungs, liver, and kidneys of mice with persistent MRSA infection and promoted M1 polarization of macrophages in the lungs. *In vitro*, BFJD decreased intracellular MRSA persisters loads and enhanced macrophage M1 polarization and M2-to-M1 repolarization. Multi-time point cellular sequencing data revealed the transcriptomic characteristics of intracellular persistent MRSA infections, including the downregulation of cytokine activity and TNF signaling pathways. GO-KEGG enrichment analysis revealed that BFJD regulated signaling pathways related to response to reactive oxygen species (ROS), IL-1β and IL-6 production, NF-κB and TNF signaling. Further intersection analysis found that genes down-regulated in the persistence state were up-regulated by BFJD, among which pro-inflammatory genes including *Il1b*, *Il*6, *Ccl2*, and *Cd40* were all reversed. Furthermore, we found BFJD enhanced the host-mediated intracellular killing of MRSA by macrophages via the CD40-ROS-NF-κB signaling cascade. Multiplex cytokine analysis showed that BFJD increased the levels of IL-1β, CCL-2, IL-6, and TNF-α in the serum of persistently infected mice. Further screening of active compounds revealed that atractylenolide II and formononetin exhibit high affinity with CD40 and decreased intracellular bacterial loads.

**Conclusion:**

BFJD decreased organ bacterial loads in mice with persistent MRSA infection by regulating the CD40-ROS-NF-κB signaling pathway, thereby modulating macrophage immunophenotypes and exerting anti-MRSA persister effects.

## Highlights

- Bufei Jiedu Formula (BFJD) effectively treats persistent MRSA infections;
- BFJD enhanced the host-mediated intracellular killing of MRSA by macrophages via the CD40-ROS-NF-κB signaling cascade;
- Multi-time point cellular sequencing data revealed the transcriptomic characteristics of intracellular persistent MRSA infections;
- Atractylenolide II and formononetin exhibit high affinity with CD40 and reduce intracellular bacterial loads.

## 1 Introduction

Methicillin-resistant *Staphylococcus aureus* (MRSA) is recognized as the leading pathogen responsible for numerous lethal infectious diseases worldwide, including pneumonia, osteomyelitis, and bacteremia (1–3). Previously, MRSA was considered an extracellular pathogen. However, MRSA has evolved the ability to persist after being taken up into host immune cells (4). The survival of MRSA within host cells provides a reservoir that is relatively protected from antibiotics, thus enabling long-term colonization of the host (5). Hospitalized patients who are colonized with MRSA are at high risk for infection after discharge (6).As an oxazolidinone antibiotic, linezolid exhibits extensive antimicrobial efficacy against Gram-positive pathogens (7). The accelerated genomic evolution of MRSA during persistent bacteremia (6.4 times faster than that of routine isolates under antimicrobial pressure) drives the emergence of multidrug resistance, including nonsusceptibility to vancomycin, daptomycin, and linezolid. This rapid adaptation, coupled with the strong selective pressure exerted by prolonged bacteremia, promotes the parallel development of both antimicrobial resistance mechanisms and bacterial persistence phenotypes, ultimately rendering clinical eradication particularly challenging (8). Although novel strategies, such as antibody–antibiotic conjugate (5), polymeric nano-system (9) and piezoelectric-enhanced sonosensitizer (10), offer innovative approaches for intracellular MRSA control, there is still a considerable distance to cover before these can be utilized clinically.

Macrophages are critical components of immune responses, capable of phagocytosis and the destruction of intracellular *S. aureus* following classical activation (10). However, *S. aureus* has developed numerous strategies to survive within, manipulate, and escape from macrophages by adapting to the phagosome environment, expressing a variety of virulence factors, or by escaping from the phagosome (11). The cycle of macrophage phagocytosis and bacterial escape can enable *S. aureus* to persist intracellularly over time (12). Notably, macrophages tend to polarize towards the M2 phenotype upon persistent MRSA infection (9). It has recently been discovered that M2 polarization correlates with increased intracellular *S. aureus* loads, increased cell death, and impaired recruitment of circulating monocytes to infection sites (13). Current studies indicate that the therapeutic strategy of repolarizing M2 macrophages into the M1 phenotype has been employed as a treatment for various cancers (14–16) or infectious diseases. The induction of cytokines is essential for macrophage polarization. Studies have also demonstrated that treatment with IFN-γ can shift macrophages from an M2 to an M1 phenotype and enhance intracellular bactericidal activity *in vitro* during MRSA infection (9). Consequently, regulating M2-associated macrophages could be a significant strategy.

The Bufei Jiedu formula (BFJD) is an effective traditional Chinese medicine prescription for long-term clinical treatment of chronic lung infections, including multi-drug resistant tuberculosis (MDR-TB). Previous clinical studies have shown that supplementing BFJD with the long-course chemotherapy regimen significantly increased the cure and cavity closure rates in MDR-PTB treatment (17). This study aims to further explore the mechanistic effects of BFJD against MRSA persistent infection. *In vivo*, a murine model of persistent MRSA infection will be used to evaluate the effects of BFJD on bacterial load in various organs and its regulation of macrophage function. *In vitro*, an intracellular MRSA persistence model will be established using macrophages, and RNA sequencing will be employed to identify core genes and signaling pathways regulated by BFJD. Finally, experimental validation will be conducted to identify and elucidate the potential targets, signaling mechanisms and active compounds of BFJD.

## 2 Methods

### 2.1 Preparation of BFJD

The Chinese formula BFJD consists of 12 herbs (17), including *Pyrola calliantha* Andres [Ericaceae] (LuXianCao, LXC) 24 g, *Prunella vulgaris* L. [Lamiaceae] (XiaKuCao, XKC) 24 g, *Scleromitrion diffusum* (Willd.) R.J.Wang (BaiHuaSheSheCao, BHSSC) 24 g, *Astragalus mongholicus* Bunge [Fabaceae] (HuangQi, HQ) 15 g, *Stemona japonica* (Blume) Miq. [Stemonaceae] (BaiBu, BB) 12 g, *Fagopyrum cymosum* (Trevir.) Meisn. (JinQiaoMai, JQM) 15 g, *Viola philippica* var. *philippica* [Violaceae] (ZiHuaDiDing, ZHDD) 9 g, *Polygonatum sibiricum* Redouté [Asparagaceae] (ZhiHuangJing, ZHJ) 9 g, *Atractylodes macrocephala* Koidz. (BaiZhu, BZ) 9 g, *Poria cocos* (Schw.)Wolf (FuLing, FL) 9 g, *Bletilla striata* (Thunb.) Rchb.f. [Orchidaceae] (Bai Ji, BJ) 3 g and *Lithospermum erythrorhizon* Siebold & Zucc. [Boraginaceae] (Zi Cao, ZC) 9 g, qualified in quality inspection according to the Chinese Pharmacopoeia. All traditional Chinese medicine (TCM) components were purchased and provided by Shanghai traditional Chinese medicine Pharmaceutical Technology Co., Ltd. All the components were extracted together two times, adding 10 and 8 times the amount of water, respectively. The extraction time was boiling for 45 minutes and 30 minutes, respectively. The extraction solution was filtered by 80-mesh sieve, and concentrated it at 60 °C to a density of approximately 1.13-1.15 mg/ml. The lyophilized powder yield was approximately 21.8%.

### 2.2 Animals

Female wild type C57BL/6J mice, aged 6–8 weeks, were procured from Charles River Laboratory Animal Technology Co. Ltd. (Zhejiang, China). The animal experiments were approved by the Institutional Animals Care and Use Committee (IACUC) of Institute Pasteur of Shanghai Chinese Academy of Science (Approval No. P2022014).

### 2.3 Chemical profiling, serum and lung tissue prototype compounds identification in the BFJD using UPLC-Q-TOF/MS

The chemical components and serum and lung tissue prototype compounds of BFJD were identified by ultra-high performance liquid chromatography coupled to quadrupole time-of-flight mass spectrometry (UPLC-Q-TOF/MS) system. To prepare the BFJD solution, 2 g of the BFJD extracts was weighed and extracted with 20 ml of 80% methanol (v/v) using ultrasonication (40 kHz) for 30 min, followed by centrifugation at 12,000 rpm for 5 min at room temperature. After extraction, the filtrate was collected using a 0.22 μm filter at a concentration of 0.1 g/ml, and subsequently, 1.0 μl of the filtrate was injected into the UPLC-Q-TOF/MS system for analysis.

Medicated and control blood samples were collected from Sprague-Dawley rats that were orally administered BFJD (2.20 g/ml, 1 ml/100 g body weight) and distilled water, respectively. Blood samples were collected at 0.5 h, 1 h, 1.5 h and 2 h post-final gavage. The samples were subsequently centrifuged at 3,000 rpm at 4°C for 10 minutes, and the upper layer of serum was collected. Methanol was added to the serum samples three times, mixed, and then centrifuged at 12,000 rpm at 4°C for 15 minutes. The supernatant was dried using nitrogen gas. The remaining residue was dissolved in 100 μl of 80% methanol (v/v) and centrifuged once more at 12,000 rpm at 4°C for 15 minutes. The collected supernatant for subsequent analysis.

For the preparation of the lung tissue solution, 1 g of control lung tissue and 5 g of medicated lung tissue were each cut into pieces and immersed in methanol at a 1:10 ratio, followed by ultrasonication for 30 min. After standing for 30 min at room temperature, the supernatant was obtained by centrifugation at 12000 rpm and 4°C about 15 min. The supernatant was then concentrated and dried to yield a residue. The residue was redissolved using 60μL and 300μL of 80% methanol (v/v), respectively, and centrifuged again at 12,000 rpm at 4°C for 15 minutes. The supernatant was then collected for subsequent analysis.

MS was performed using an AB Sciex Triple TOF^®^ 4,600 (AB SCIEX, Foster City, CA, USA) instrument to identify the BFJD solution. The optimized MS parameters were as follows: ion source temperature: 500°C; curtain gas: 35 psi; ion source gas 1 and 2: 50 psi; ion spray voltage: 5000 V (positive), 4500 V (negative); declustering potential: 100 V (MS and MS/MS); collision energy 10 eV; collision energy spread 20eV (MS/MS); mass range: 100–1500 m/z (MS), 50–1700 m/z (MS/MS); ion release delay: 30 ms; ion release width: 15 ms. Subsequently, to identify the serum and lung tissue prototype compounds in the BFJD, the Agilent 6545 Q-TOF (Agilent, Santa Clara, California, USA) was employed with the following operational parameters: gas temperature, 320°C; drying gas, 8 l/min; nebulizer, 35 psi; shealth gas temperature, 350°C; shealth gas flow, 11 l/min; Vcap, 4000 V; nozzle voltage, 1000 V; Fragmentor, 175 V; skimmer, 60 V; collision energy spread 20eV (MS/MS); mass range, 50–1700 m/z (MS and MS/MS).

### 2.4 Bacterial strains

The MRSA strain ST239 (18) was a generous donation from Professor Wenhong Zhang from Huashan Hospital Affiliated to Fudan University. The bacteria were cultured in Luria-Bertani (LB) medium at 37°C with shaking at 200 rpm and harvested by centrifugation at 3000 rpm for 5 min during the Mid-Log phase. The bacterial pellet was then washed twice with sterile phosphate-buffered saline (PBS). Subsequently, the bacterial culture was resuspended in PBS, and the colony forming units (CFU) were adjusted to the desired concentration using a Turbidimeter (BioMérieux).

### 2.5 Chronic infection model

The mice were inoculated with 1×10^8^ CFU of MRSA in 0.2 ml of PBS via a lateral tail vein. Twenty-eight days post-infection, the mice were divided into three groups: the Model group, the BFJD group, and the Linezolid group. After 14 days of treatment, the mice were euthanized and sampled. The bacterial load in the lungs, liver, and kidneys was determined by preparing organ homogenates in sterile PBS and plating 10-fold serial dilutions on LB agar.

The clinical dosage for an adult weighing 70 kg of BFJD is 162 g (crude drug) daily. Based on the body surface area index and lyophilized powder yield (21.8%), the animal medium dosage (4.59 g/kg/d) of BFJD is calculated to be equivalent to the clinical dosage. Linezolid was purchased from Abmole (HOU, USA) and dissolved in dimethyl sulfoxide (DMSO) with a final solution volume not exceeding 0.5%, and subsequently diluted in sterile water. The final oral concentration was 50mg/kg/d (19).

### 2.6 Cell viability assay

RAW264.7 macrophages were seeded into a 96-well plate at a density of 1×10^4^ cells per well. A blank group was included as a background control. After the cells adhered fully, the previous culture medium was removed, and different concentrations of BFJD solution (50-800 μg/mL) and active compounds (0-200 μM), prepared in DMEM complete medium, were added for 24 or 48 hours. Following the intervention, the culture medium was removed, and CCK-8 reagent (Beyotime, Shanghai, China) was added. The cells were incubated for 30–60 minutes, and the absorbance at 450 nm was measured using a microplate reader to calculate cell viability.

### 2.7 Intracellular antibacterial experiments of MRSA persisters

The macrophage intracellular MRSA persistence infection model was established (5, 9) to examine the impact of BFJD on macrophage-mediated clearance of intracellular MRSA. RAW264.7 macrophages were seeded at 2×10^5^ cells per well. The multiplicity of infection (MOI) was set at 20, and the phagocytosis period was established at 1 hour. Extracellular residual MRSA was eliminated through treatment with lysostaphin. The cells were then cultured for 24 hours in a CO_2_ incubator to mimic the intracellular MRSA colonization environment. Subsequently, the corresponding concentrations of BFJD solution (20, 21), IFN-γ, and linezolid were added to the wells containing DMEM infection medium. The cells were cultured for an additional 24 hours. The experimental groups consisted of: model group, BFJD group (100 μg/mL, 200 μg/mL, 400 μg/mL), linezolid group (6 μg/mL), IFN-γ group (40 ng/mL), with three replicates for each group.

### 2.8 Flow cytometry analysis

For the MRSA infection animal experiment, the left lung lobe was minced and digested with 50 μg/ml Liberase TM (Roche) and 1 μg/ml DNase I (Sigma) for 45 min at 37°C. Subsequently, cells were surface-stained for 30 minutes on ice with experimentally designed combinations of the following antibodies: Live-Dye eFluor™ 780e, CD45-BV510, CD11b-PE, F4/80-FITC, CD80-Brilliant Violet 421™, and CD206-APC. After staining, the cells were fixed, and flow cytometry was performed using a Beckman CytoFlex S (Beckman, USA) with CytExpert Software (v.2.0).

For the intercellular MRSA persistent infection experiment, antibodies selected were Live-Dye eFluor™ 780e, CD80-Brilliant Violet 421™, and CD206-APC. The experimental cells were collected, washed, stained, fixed, and analyzed within 48 hours using flow cytometry.

For the bone marrow-derived macrophages (BMDM) repolarization experiment, bone marrow-derived monocytes were extracted from wild-type mice and differentiated into macrophages using M-CSF (20 ng/mL). To induce M2 polarization, IL-4 and IL-13 (100 ng/mL) were added. The flow cytometry antibodies used were Live-Dye eFluor™ 780e, CD11b-PE, F4/80-FITC, CD80-Brilliant Violet 421™, and CD206-APC. The experimental cells were collected, washed, stained, fixed, and analyzed within 48 hours using flow cytometry.

### 2.9 RNA sequencing analysis and visualization

Following the establishment of persistent MRSA infection, cells were sampled at different time points—1 hour, 24 hours, and 48 hours post-intracellular infection—and the concentration of the BFJD intervention was set at 400 μg/ml during the 24–48 hours of intracellular infection. Per condition, four biological replicates were performed. Total RNA was extracted by Trizol Reagent, and the RNA quality was assessed using Qubit^®^3.0 Fluorometer (Life Technologies, CA, USA) and Nanodrop One spectrophotometer (Thermo Fisher Scientific Inc, USA). To establish an RNA library, the mRNA polyA-based enrichment method was performed. The RNA samples were sequenced using DNBSEQ-T7 at the Wuhan BGI Technology Service Co. Ltd. Quality-filtered RNA-seq reads were aligned to the mouse genome (GRCm38/mm10; https://useast.ensembl.org/index.html) by STAR software (version 2.7.4). Principal component analysis (PCA) was performed based on the raw count values of genes from each sample and visualized. The raw count values for each gene were then converted into normalized FPKM (Fragments Per Kilobase of exon per Million fragments mapped) values. Differentially expressed genes were identified using the DESeq2 (Ver. 1.36.0) package with a threshold of fold change (FC ≥ 1.5, adj p-value ≤ 0.05). Volcano plots were generated to illustrate these genes. Venn diagram was drawn to visualize the differentially expressed gene clusters between groups, and the reversed genes were also identified and visualized. Gene Ontology (GO) and KEGG (Kyoto Encyclopedia of Genes and Genomes) pathway enrichment analysis of the gene clusters were performed using the DAVID online tool, and the bubble plots were generated. All visualizations were performed using R software (Ver. 4.2.1).

### 2.10 Quantification of cytokines

Blood samples were collected and centrifuged at 1200×g for 10 min to isolate serum. Multiple cytokines in mouse serum were measured using the Biolegend Multiplex kit (Biolengend, CA, USA) according to the manufacturer’s instructions. Flow cytometric detection was performed, and the results were analyzed and exported using the Biolegend LEGENDplex™ QOGNIT platform (https://legendplex.qognit.com/).

### 2.11 Detection of intracellular reactive oxygen species/mitochondrial superoxide

ROS levels were detected using ROS Assay Kit (Beyotime, Shanghai, China) according to the manufacturer’s instructions. The fluorescence intensity was analyzed using flow cytometry or a fluorescence microscope. MitoSoX was detected using the MitoSoX Assay Kit with MitoSoX™ Red (Beyotime, Shanghai, China), also following the manufacturer’s instructions. The fluorescence intensity was analyzed by flow cytometry.

### 2.12 CD40 gene knockdowm assay

BMDM cells were transfected with the CD40 siRNA-mate plus kit (GenePharma, Shanghai, China) according to the manufacturer’s instructions. To elaborate, BMDMs were treated with 30 pmol of CD40 siRNA in serum-free medium containing the transfection reagent for 48 hours. The efficiency of CD40 expression knockdown was subsequently verified through flow cytometry analysis (22). The CD40-silenced BMDMs were then subjected to MRSA infection using the established experimental protocol. **Supplementary Table S1** provides the siRNA sequence for gene knockdown.

### 2.13 Luciferase assay

RAW264.7 cells stably expressing NF-κB-dependent SEAP were constructed as previously described (23). Following the establishment of persistent MRSA infection, BFJD solution was combined with the NF-κB inhibitor BAY-11-7082 (10 μM) and ROS inhibitor NAC (5 mM) and added to the wells. BAY 11–7082 and NAC were purchased from MedChemExpress (NJ, USA). The cells were then cultured for an additional 24 hours. NF-κB activity was assessed using the Firefly Luciferase Reporter Gene Assay Kit (Beyotime Biotechnology, shanghai, China). Cells were lysed with the passive lysis buffer provided in the kit. Subsequently, 50ul of luciferase detection solution was added to the supernatant of each well with a BeyoGold™ 96 well microplate (Beyotime Biotechnology), and detection was performed on a full wavelength multifunctional microplate detector (Infinite^®^ E Plex, TECAN, Switzerland).

### 2.14 Immunofluorescence detection

To determine the macrophage subtypes in the lungs, we conducted immunofluorescence staining on lung tissue sections. The process involved a series of standardized procedures, including tissue sectioning, dewaxing, antigen retrieval, endogenous peroxidase blockade, and serum blocking. Subsequently, the lung tissue sections were incubated with primary antibodies against CD68 (BA3638, Boster, 1:200), CD86 (CY5238, ABways, 1:200) and CD206 (PTG, 60143-1-Ig, 1:200) overnight at 4°C. The slices were then further incubated with HRP-conjugated goat anti-rabbit IgG for 60 minutes at 37°C, followed by fluorescein-conjugated anti-HRP for 30 minutes at 37°C. In this process, CD68 corresponds to TYR-555, CD86 to TYR-488, and CD206 to TYR-651. The nuclei were counterstained with DAPI (28718-90-3, Sigma, 1:1000) for 60 minutes at room temperature. Images were captured using an inverted fluorescence microscope (Leica, Germany).

### 2.15 Molecular docking and visualization

Molecular docking simulations were performed using the AutodockTools (v.1.5.6) and Autodock Vina software (v.4.2). The structure of active compounds were downloaded from the Pubchem database, and then hydrogenated by AutodockTools. The structure CD40 (PDB ID: 5dmi) were obtained from the RCSB (https://www.pdb.org/) and introduced into AutodockTools for dehydration and hydrogenation, facilitating ligand separation. Autodock Vina was utilized to conduct molecular docking of target proteins with active compounds and to calculate their free binding energies. Results with binding energies lower than -5 kcal/mol were selected for analysis of local binding conformations and hydrogen bonding interactions using Discovery Studio Visualizer (v. 25.1) software.

### 2.16 Statistical analysis

The Student’s t-test or one-way analysis of variance (ANOVA) was employed for statistical analysis, and differences between groups were compared. Statistical analysis and graphs were generated using GraphPad Prism 8.0 software (GraphPad Software Inc., San Diego, CA, USA). p-value of less than 0.05 was considered statistically significant.

## 3 Results

### 3.1 BFJD promote the clearance of persistent MRSA infection and macrophage M1 polarization *in vivo*

**Figure 1:**
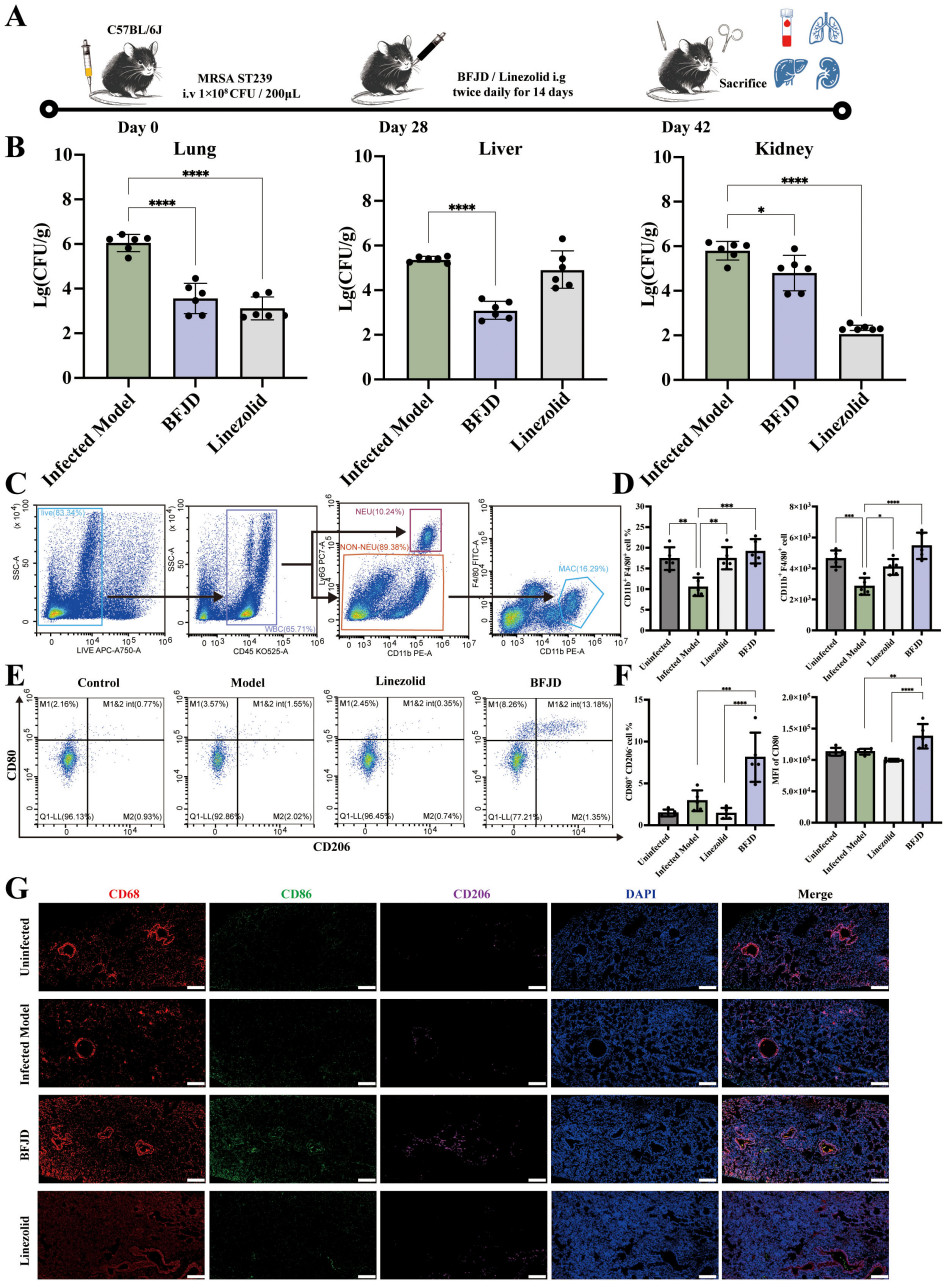
BFJD promotes the clearance of persistent MRSA infection and macrophage M1 polarization *in vivo*. **(A)** Schematic illustration of a 42-day long-term persistent MRSA infection model. Mice were inoculated with 1×10^8^ CFU of MRSA in 0.2 ml of PBS via a lateral tail vein. At day 28 post- MRSA inoculation, mice were administered BFJD and linezolid by oral gavage intervention, and after 14 days of treatment, the mice were euthanized for sampling. **(B)** Bacterial burdens in the lungs, liver, and kidneys of mice (n=6). **(C)** The gating strategy of macrophages (CD11b+F4/80+) in mouse lung tissue flow cytometry. **(D)** The percentage of macrophages in the Ly6G - cell population and relative count in total harvested cells (n=5). **(E)** Representative FACS plots of macrophage polarization. **(F)** The percentage of M1- type (CD80+CD206-) macrophages and MFI of CD80 (n=5). **(G)** Representative immunofluorescence micrograph of lung tissue stained with indicated antibodies for CD68, CD86 and CD206. Nuclei were revealed by DAPI staining. Scale bars, 200 μm. Data are presented as the mean ± SD. Differences were analyzed applying ordinary one-way ANOVA followed by Dunnett´s multiple comparisons test (comparison with the Infected Model). *P < 0.05, **P < 0.01, ***P < 0.001, ****P < 0.0001.

To explore the antibacterial effect of BFJD, we initially established acute and subacute infection model with 1×10^8^ CFU of MRSA in 0.2 ml of PBS via a lateral tail vein (**Supplementary Figure S1A**). As shown in **Supplementary Figure S1B**, regardless of whether in the clinical equivalent dose of BFJD (4.59 g/kg/d) or the clinical double dose of BFJD (9.18 g/kg/d) group, BFJD intervention from day 0 to day 4 after infection, and BFJD intervention from day 4 to day 12 after infection, the bacterial load did not show a significant reduction in the lung, liver and kidney, compared to the model group. Given that BFJD did not show obvious antibacterial effects in acute and subacute infection, a murine model of persistent MRSA infection were established to further validate its effects in persistent infection (**Figure 1A**). In contrast to acute and subacute infection, the bacterial load in the lungs, liver, and kidneys were significantly decreased after the treatment of BFJD, suggesting that BFJD may exert certain antibacterial effects in the MRSA persistent infection (**Figure 1B**).

To further investigate the antibacterial mechanism of BFJD, flow cytometry analysis of lung cell populations from MRSA persistent infection mice was performed to verify the immune regulation of BFJD. The gating plots and scatter plots were shown in **Figures 1C, E**.The statistical results (**Figure 1D**) indicated that the proportion of macrophages in mice with persistent MRSA infection was reduced compared to uninfected mice, which may be associated with macrophage death mediated by bacteria during persistent infection (24, 25). However, both BFJD and linezolid increased the proportion and quantity of macrophages when compared with infected mice. Furthermore, BFJD increased the proportion of M1 (CD80^+^CD206^−^) macrophages and enhanced the mean fluorescence intensity (MFI) of the M1 surface marker CD80 (**Figure 1F**). Given the immune regulatory effects of BFJD on macrophages in MRSA persistent infection, an immunofluorescence assay was performed for further verification. As shown in the **Figure 1G**, the green fluorescence (CD86) was increased upon BFJD intervention, suggesting that BFJD increased the proportion of M1 in the lung tissue. In conclusion, these results suggest that BFJD may exert its antibacterial effect in MRSA persistent infection through macrophage-mediated immune regulation.

### 3.2 BFJD promotes the clearance of MRSA persisters and induces macrophage M1 polarization and M2-to-M1 repolarization *in vitro*

**Figure 2:**
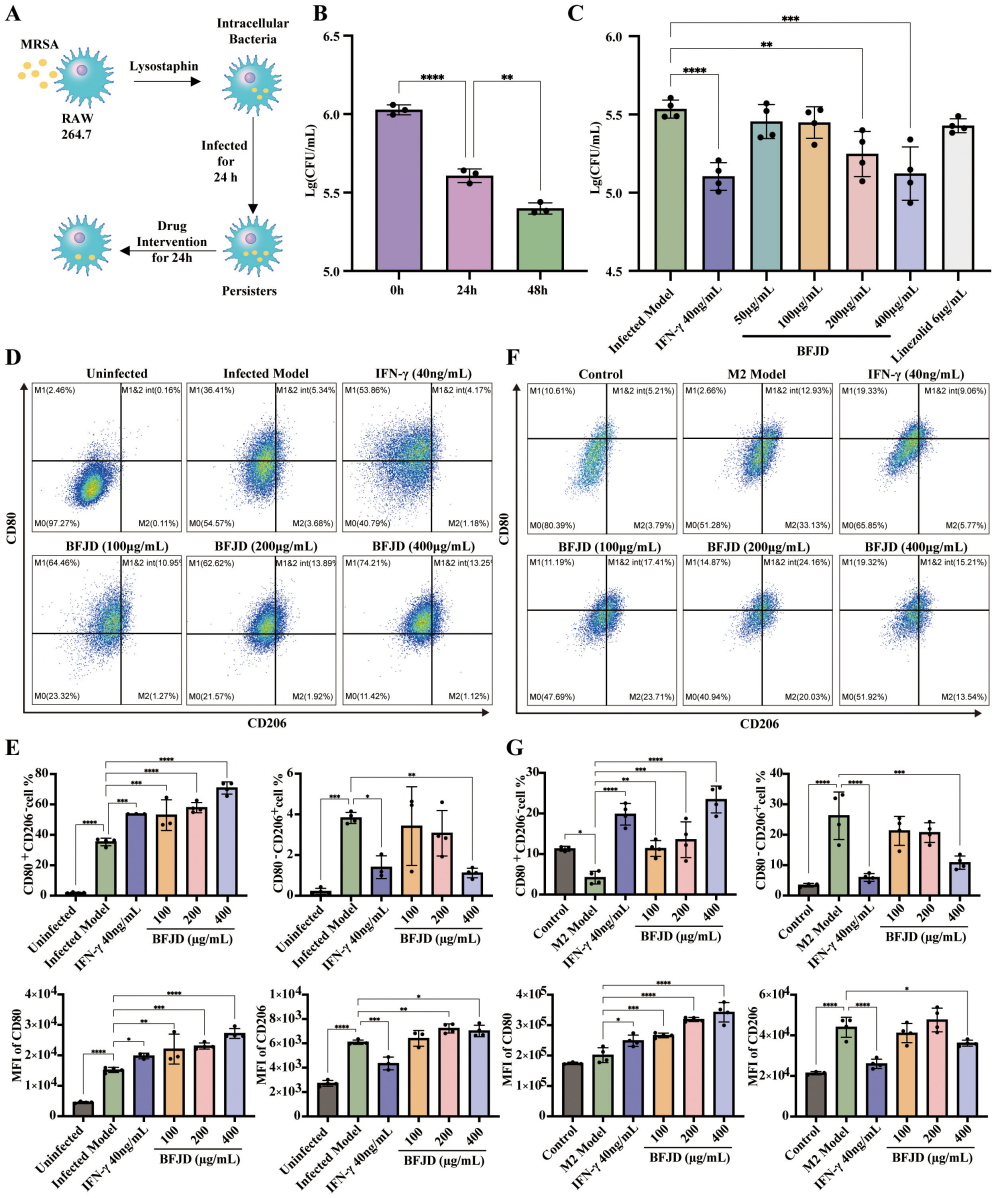
BFJD enhances the clearance of intracellular MRSA persister and promotes macrophage M1 polarization and M2-to-M1 repolarization *in vitro*. **(A)** Schematic illustration of the intracellular MRSA persister model and drug intervention in RAW264.7. **(B)** Dynamics of intracellular bacterial burden at 0h, 24 hour post-incubation (hpi) and 48 hpi (n=3). **(C)** Killing effect of different concentrations of BFJD, IFN-γ, and linezolid on intracellular MRSA persister (n=4). **(D)** Representative FACS plots showing macrophage polarization in intracellular MRSA persister infection at 48 hpi. **(E)** The percentage and MFI of M1 (CD80+CD206-) and M2 (CD80-CD206+) macrophage types in intracellular MRSA persister infection at 48 hpi (n=3-4). **(F)** Representative FACS plots of macrophage polarization in IL-4 and IL-13 induced M2 polarization. **(G)** The percentage and MFI of M1 (CD80+CD206-) and M2 (CD80-CD206+) macrophage types in IL-4 and IL-13 induced M2 polarization (n=3-4). Data are presented as the mean ± SD. Differences were analyzed applying ordinary one-way ANOVA followed by Dunnett´s multiple comparisons test comparison with 24 h for **(B)** and the Infected Model for **(C, E, G)**. *P < 0.05, **P < 0.01, ***P < 0.001, ****P < 0.0001.

Furthermore, we investigated the killing effect of BFJD on MRSA persister. Initially, we verified the direct activity of BFJD on MRSA growth inhibition through minimal inhibitory concentration (MIC) determination. The results showed that BFJD, even at a concentration of 1600 μg/mL, exhibited no direct inhibitory effect on MRSA. To simulate the MRSA persistent infection within macrophages, we adopted a persistent infection cell model based on previous studies (5, 9), which eliminates extracellular MRSA through lysostaphin (**Figure 2A**). At 0 h, 24 h, and 48 h post-infection, macrophages were lysed, and the intracellular bacterial load was quantified. The results showed that, although macrophages partially cleared intracellular MRSA over 48 hours, persistent colonization remained, confirming the limitations of macrophages in controlling persistent infection (**Figure 2B**). Our finding, illustrated in **Supplementary Figure S2**, demonstrated that intervention with BFJD exhibited no cytotoxicity toward macrophages after 24 h or 48 h at concentrations ranging from 0 to 800 μg/mL. Subsequently, We treated macrophages with different concentrations of BFJD for 24 h and quantified the intracellular bacterial load. As depicted in **Figure 2C**, the intracellular bacterial burden was significantly reduced by BFJD at concentrations of 200 μg/mL and 400 μg/mL, as well as by IFN-γ (40 ng/mL). In contrast, linezolid did not promote killing of intracellular MRSA persister, not even at 3×MIC. Meanwhile, flow cytometry was performed to analyze the polarization of macrophage. The results showed that the proportion of M1 was promoted by BFJD at 100 μg/mL, 200 μg/mL, and 400 μg/mL, as well as IFN-γ (40 ng/mL), also indicated by a rightward shift in the CD80 overlay histogram (**Figures 2D, E**). Furthermore, the proportion of M2 (CD80^−^CD206^+^) was reduced by BFJD at 400 μg/mL and IFN-γ at 40 ng/mL (**Figures 2D, E**). These findings suggest that BFJD may exert its intracellular antibacterial effects by promoting M1 macrophage polarization, which is consistent with *in vivo* experiments.

Considering that M2 macrophage polarization may contribute to immunosuppressive effects in the context of MRSA persistent infection, we established a M2-polarized bone marrow-derived macrophage (BMDM) model to verify the effect of BFJD on macrophage repolarization. The results demonstrated that the proportion of M1 macrophages was increased by BFJD at 100 μg/mL, 200 μg/mL, and 400 μg/mL, as well as IFN-γ (40 ng/mL), also evidenced by a rightward shift in the CD80 overlay histogram. Additionally, the proportion of M2 was reduced by BFJD at 400 μg/mL and IFN-γ at 40 ng/mL, also evidenced by a leftward shift in the CD206 overlay histogram (**Figures 2F, G**). In summary, these findings suggest that BFJD promotes M1 polarization and M2-to-M1 repolarization under infection or cytokine-induced conditions, contributing to its potential effects in enhancing macrophage-mediated persistent bacterial clearance.

### 3.3 BFJD mediates the regulation of pro-inflammatory gene expression

To further elucidate the mechanisms by which BFJD promotes the clearance of persistent MRSA infection in macrophages, RNA-seq analysis was conducted to comprehensively profile gene expression and functional alterations. Based on the results in **Figures 2C–E**, we observed that BFJD exhibited an optimal regulatory effect on macrophages at a concentration of 400 μg/mL, promoting the polarization of macrophages to clear intracellular bacteria. Consequently, this concentration was selected for RNA-seq analysis and subsequent experiments.

**Figure 3:**
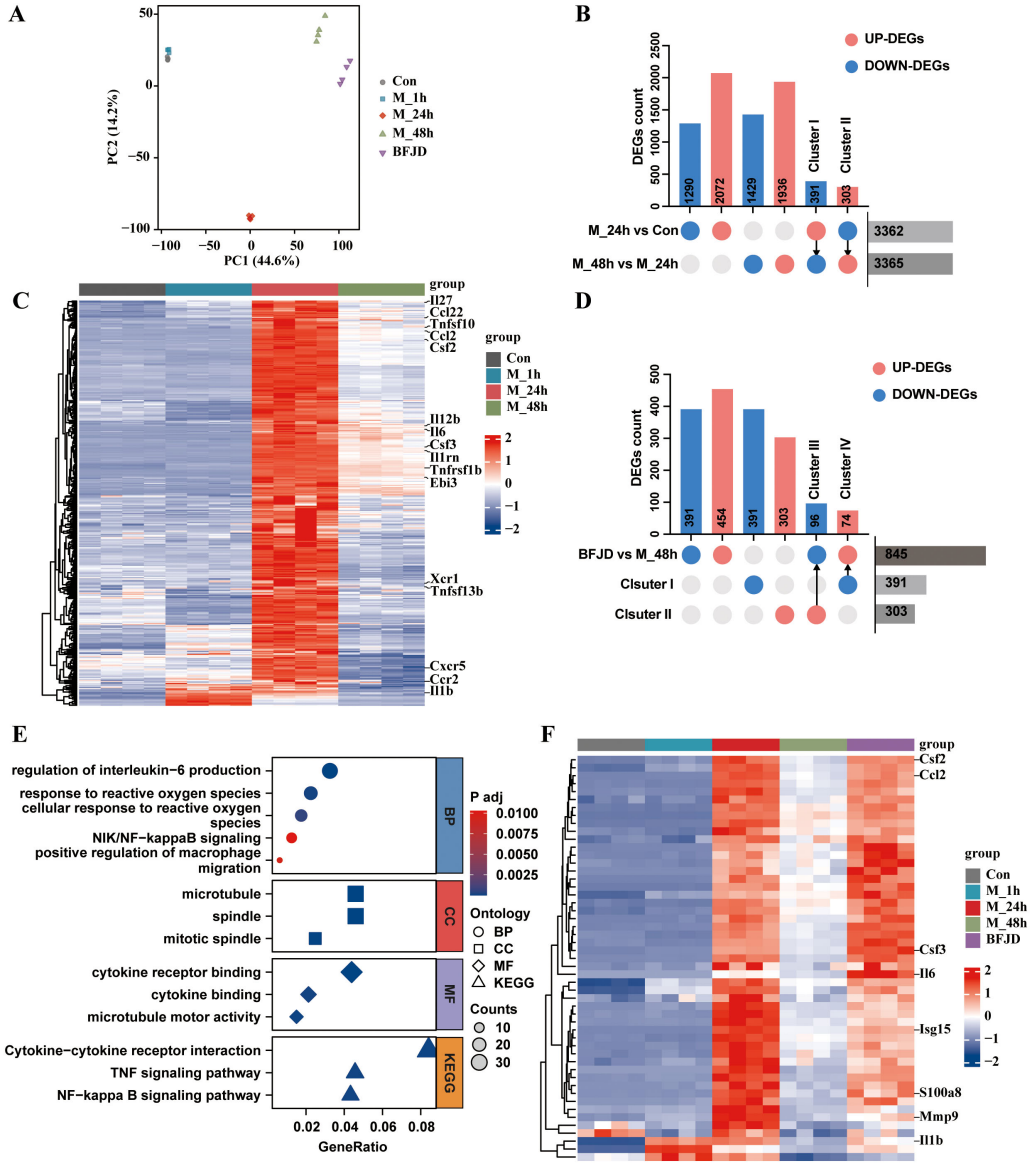
Analysis and verification of the transcriptome profile regulated by BFJD in intracellular MRSA persister infection *in vitro*. **(A)** Classification results of uninfected control (Con), intracellular MRSA infection after 1 hour (M_1h), intracellular MRSA infection after 24 hour (M_24h), intracellular MRSA infection after 48 hour (M_48h), and BFJD groups (n=4) based on PCA score plot. **(B)** Distributions of DEGs between two groups (“M_24h vs Con” and “M_48h vs M_24h”) and the reversed DEGs. **(C)** Heatmap of the gene cluster I (391 DEGs) (FC ≥ 1.5, adj p-value ≤ 0.05). **(D)** Distributions of DEGs between two groups (BFJD vs M_48h) and the reversed DEGs compared with Cluster I or Cluster II. **(E)** GO and KEGG enrichment analysis of the DEGs from “BFJD vs M_48h”. **(F)** Heatmap of the cluster IV (DEGs reversely expressed by BFJD) (FC ≥ 1.5, adj p-value ≤ 0.05).

PCA indicated that all samples were within the 95% confidence interval, suggesting high within-group consistency and significant differences between groups (**Figure 3A**). A total of 3,362 differentially expressed genes (DEGs) were identified between 24h post-intracellular infection and the control, with 2,072 up-regulated and 1,290 down-regulated. Furthermore, 3,365 DEGs were determined between 48h post-intracellular infection compared to 24h post-intracellular infection, with 1,936 up-regulated and 1,429 down-regulated. DEGs were visualized using volcano plot (**Supplementary Figures S3A, B**). Intersection analysis revealed gene Cluster I, which consists of 391 reversed genes that were up-regulated at 24h but down-regulated at 48h post-intracellular infection. This cluster includes the down-regulation of inflammatory genes such as *Il1b, Il6*, *Ccl2*, etc (**Figures 3B, C**). Concurrently, GO-KEGG enrichment analysis of Cluster I further demonstrated that bacterial persistence down-regulated innate immune response, cytokine receptor binding, cytokine activity, and TNF signaling pathways (**Supplementary Figure S3D**).

To further understand the effect of BFJD on the transcriptome of macrophage with persistent infection, DEGs analysis was conducted between BFJD group and M_48h group. The DEGs were visualized using a volcano plot (**Supplementary Figure S3C**). Following BFJD intervention, 454 genes were upregulated and 391 genes were downregulated (**Figure 3D**). GO&KEGG enrichment analysis further revealed that BFJD regulated IL-1β and IL-6 production, response to ROS, NF-κB and TNF signaling pathways (**Figure 3E**), suggesting that these pathways may contribute to its intracellular antibacterial effects. Upon visualizing genes within the three most up-regulated KEGG pathways, we observed that BFJD significantly boosted the expression of M1 macrophage polarization markers, such as *Cd40*, *Ccr2*, *Cxcl2*, *Tnf*, and *Il12b*. The data also revealed a coordinated chemotactic response, evidenced by the simultaneous upregulation of chemokine receptors (*Ccr1/2*, *Cxcr3*) and their ligands (*Ccl2/3/4/5*, *Cxcl2/3*) (**Supplementary Figure S3E**). Intersection analysis was performed to align DEGs regulated by BFJD with Cluster I or Cluster II, which were defined as reversed genes. Subsequently, gene Clusters III (96 genes) and IV (74 genes) were identified as the key genes that were up-regulated or down-regulated by BFJD, respectively (**Figure 3D**). By screening these key genes, we found that the pro-inflammatory genes, which were suppressed in the persister state, were concentrated in Cluster IV. Through a heatmap of Cluster IV, we observed that these pro-inflammatory genes, including *Il1b, Il6*, *Ccl2* and *Cd40*, were all restored after BFJD intervention (**Figure 3F**). In summary, these results indicate that MRSA persistent infection reprograms the host transcriptome, including the pro-inflammatory response and innate immunity, while BFJD may exert antibacterial effects by restoring these inflammatory responses in macrophages.

### 3.4 BFJD mediates the CD40-ROS-NF-κB signaling pathway to eliminate MRSA persister

**Figure 4:**
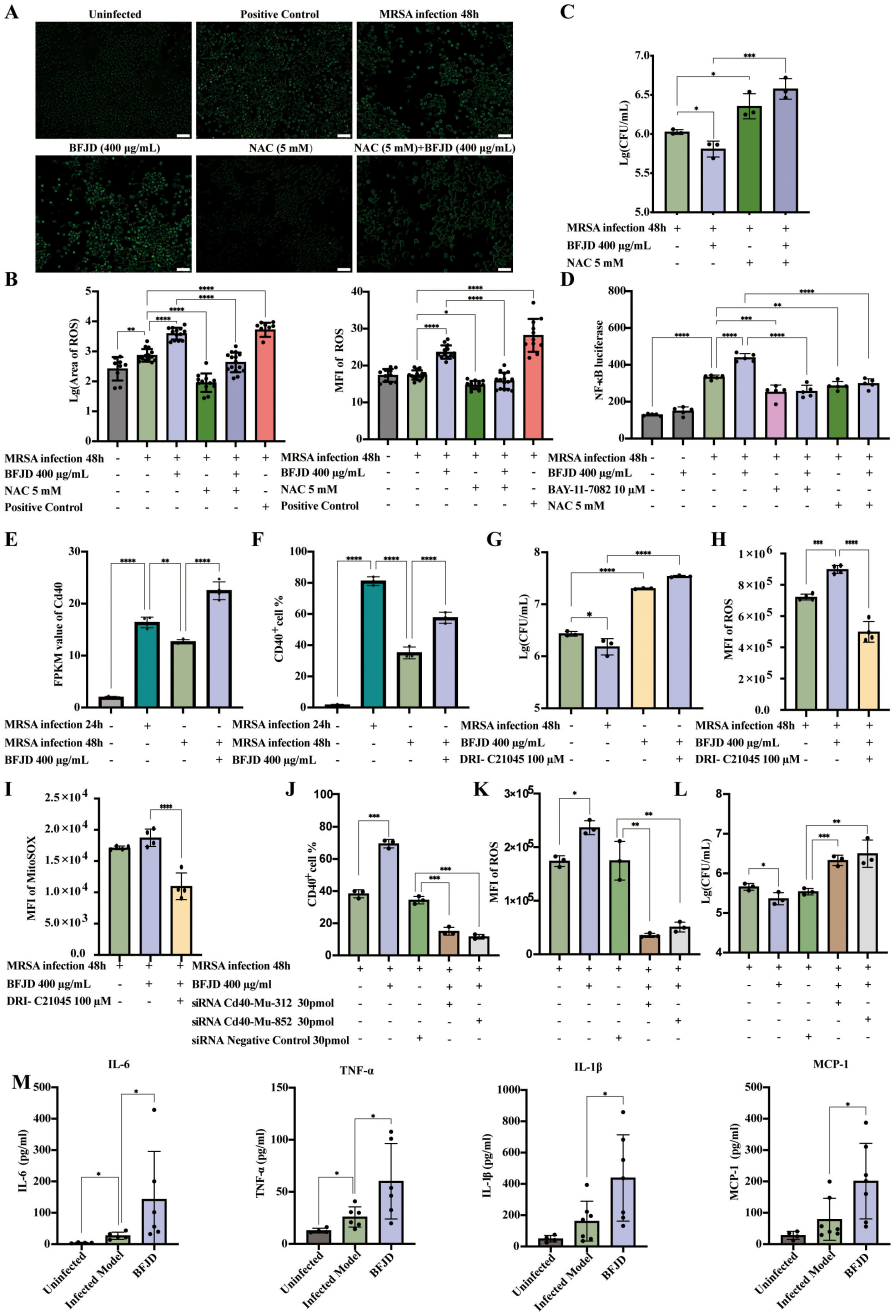
BFJD mediates CD40-ROS-NF-κB signaling pathway to eliminate MRSA persister cells. **(A, B)** Representative images and quantitative analysis of intracellular ROS in RAW264.7 (n=11-15). **(C)** Intracellular bacterial burden at 48 hpi (n=3). The ROS inhibitor NAC was used to observe its effect on the intracellular antibacterial activity of BFJD. **(D)** Relative luciferase activity in RAW264.7 cells stably expressing NF-κB-dependent SEAP (n=5). ROS inhibitors NAC were used to observe their effects on the regulation of NF-κB activity by BFJD. The NF-κB inhibitor BAY-11–7082 was used as a negative control. **(E)** CD40 expression in the cellular transcriptomes (n=4). **(F)** CD40 expression were detected by flow cytometry in RAW264.7 (n=3). **(G)** Intracellular bacterial burden at 48 hpi. The CD40 signaling inhibitor DRI-C21045 was used to observe its effect on the intracellular antibacterial activity of BFJD (n=3). **(H)** Detection of intracellular ROS in infected model, BFJD and BFJD combined with the CD40 signaling inhibitor DRI-C21045 (n=4). **(I)** Detection of intracellular mitochondrial superoxide in the infected model, BFJD and BFJD combined with the CD40 signaling inhibitor DRI-C21045 (n=4). **(J)** CD40 gene were knockdown by siRNA, CD40 expression were detected by flow cytometry in BMDMs (n=3). **(K)** ROS were detected by flow cytometry in BMDMs (n=3). **(L)** Intracellular bacterial burden at 48 hpi (n=3). Figure **(J-L)** share the same head title. **(M)** The concentrations of MCP-1, IL-6, TNF-α and IL-1β in the serum of uninfected (n=4), infected model (n=6-7), BFJD (n=6-7) groups at 42 days post infection (dpi). Data are presented as the mean ± SD. Differences were analyzed applying ordinary one-way ANOVA followed by Dunnett´s multiple comparisons test comparison with MRSA infection 48h for **(B-G)**, MRSA infection 24h for **(E, F)**, BFJD 400 μg/ml for **(G-L)**, siRNA NC group for **(J-L)** and the Infected Model for **(M)**. *P < 0.05, **P < 0.01, ***P < 0.001, ****P < 0.0001.

Redox regulation is associated with the immune-inflammatory response and M1 polarization (26). Based on GO analysis, we found that ROS, an effective antibacterial substances, may be closely associated with the immune effect of BFJD. To further investigate the effect of BFJD on ROS, we examined ROS expression using fluorescence microscopy (**Figure 4A**). The results indicated that MRSA infection could increase the intercellular ROS level, while BFJD further enhanced ROS production. In contrast, N-acetylcysteine (NAC), an ROS inhibitor, either alone or in combination with BFJD, reduced the ROS level (**Figure 4B**). Additionally, NAC treatment significantly promoted bacterial load and negatively impacted the antibacterial effect of BFJD (**Figure 4C**), suggesting that BFJD may enhance its antibacterial activity by promoting ROS production. As a critical intracellular signaling molecule, ROS plays a key role in regulating inflammatory signaling. Previous studies have demonstrated that ROS regulation of the NF-κB signaling pathway is essential for antibacterial immunity (27).

Thus, we employed RAW264.7 cells that stably express an NF-κB reporter gene to evaluate the effect of BFJD on NF-κB activity, using the NF-κB inhibitor BAY-11–7082 as a negative control. As shown in the **Figure 4D**, BFJD did not elicit NF-κB reporter gene expression in uninfected cells. However, NF-κB reporter gene expression was markedly increased by MRSA infection, and BFJD further augmented this expression. Moreover, NAC, whether administered alone or in conjunction with BFJD, inhibited NF-κB reporter gene expression, indicating that BFJD might modulate antibacterial immunity via the ROS-NF-κB signaling pathway.

To identify the key target through which BFJD activates the ROS-NF-κB pathway *via* cell membrane surface proteins, we analyzed the DEGs under BFJD intervention (22, 28, 29), hypothesizing that the transmembrane protein CD40 might be a critical target in macrophage-mediated clearance of intercellular MRSA infection. Transcriptomic data indicated that the expression of CD40 was significantly reduced at 48hpi compared to 24hpi. Notably, BFJD intervention substantially restored CD40 expression (**Figure 4E**), a finding corroborated by flow cytometric analysis of CD40^+^ macrophages (**Figure 4F**). Therefore, the CD40 inhibitor DRI-C21045 was applied for further validation. The bacterial load increased and negatively impacted the intracellular antibacterial effect of BFJD upon treatment with DRI-C21045 (**Figure 4G**), indicating that CD40 plays a crucial role in BFJD-mediated immune regulation. As a cell surface receptor, CD40 is known to positively regulate multiple inflammatory signaling pathways, including the ROS pathway (30, 31).Therefore, total ROS and MitoSoX levels were detected by flow cytometry. As shown in the **Figures 4H, I**, BFJD significantly increased the MFI of both total ROS and MitoSoX compared to the MRSA infection group, with the overlapping histograms in **Supplementary Figures S4A, B**; however, these effects were reversed by treatment with DRI-C21045. Furthermore, genetic validation through siRNA-mediated CD40 knockdown in BMDMs further confirmed these findings. As clearly demonstrated in **Figure 4J**; **Supplementary Figure S4C**, CD40 silencing abrogated BFJD-induced upregulation of CD40, along with ROS production (**Figure 4K**; **Supplementary Figure S4D**) and bacterial clearance (**Figure 4L**). These consistent results from both pharmacological inhibition and genetic knockdown establish CD40 as the crucial membrane target to enhance macrophage-mediated bacterial clearance. Next, several cytokines and chemokines in persistent infection mouse serum were detected by LEGENDplex or ELISA analysis. The results demonstrated that BFJD significantly upregulated the serum levels of IL-1β, CCL-2, IL-6, and TNF-α (**Figure 4M**). Taken together, these results suggest that BFJD exerts its antibacterial effect against MRSA persistent infection through the CD40-ROS-NF-κB signaling pathway.

### 3.5 Bioactive compounds from BFJD targeting CD40 to exert antibacterial effect

Given the complex constituents in TCM ingredients, the characterization of components in BFJD was achieved through UPLC-Q-TOF/MS. The chemical base peak ion chromatogram of BFJD exhibits distinct profiles in both negative and positive ion modes, as shown in **Supplementary Figures S5A, B**, respectively. A total of 115 chemical compounds were tentatively characterized within BFJD. **Supplementary Table S2** provides comprehensive data. Furthermore, prototype compounds identified in the bloodstream and lung tissues from BFJD were selected to explore their potential as bioactive agents for targeting CD40 to exert antibacterial effect. By consulting the Natural Products HR-MS/MS Spectral Library database and relevant literature (32), a total of 26 prototype compounds were discovered, with 18 entering the bloodstream, 21 reaching the lungs, and 13 components entering both the bloodstream and the lungs. **Supplementary Table S3** illustrates detailed information of the 26 compounds, and 20 commercially available compounds were selected for subsequent investigations (**Supplementary Table S4**).

**Figure 5:**
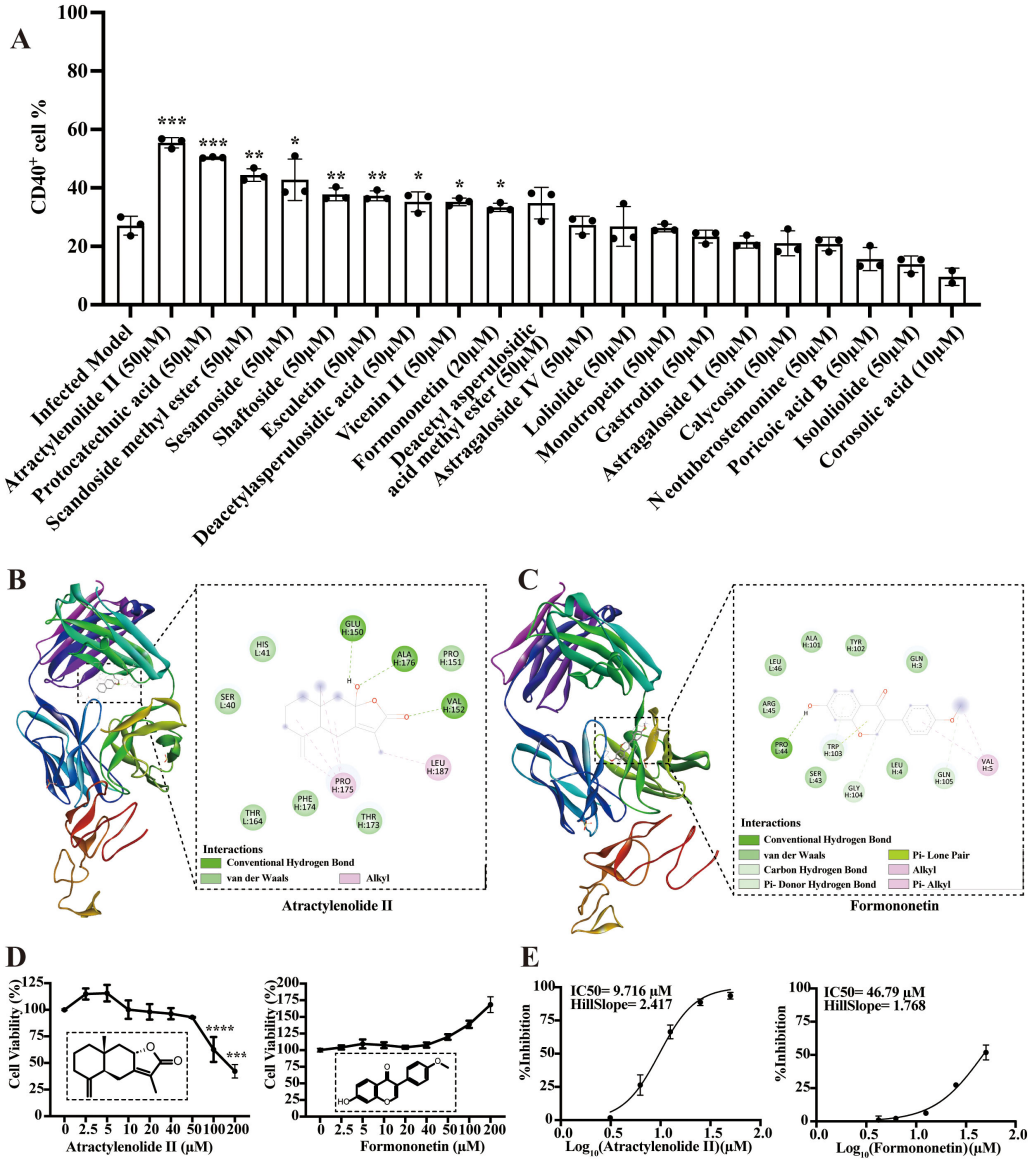
Bioactive compounds from BFJD targeting CD40 to exert antibacterial effect. **(A)** The impact of 20 active compounds in BFJD on CD40 expression was assessed using flow cytometry in a macrophage intracellular MRSA persistence infection model (n=3). **(B, C)** Overview of the structures of atractylenolide II- and formononetin-bound CD40, presented in 2D and 3D representations. **(D)** Cell viability curves of atractylenolide II and formononetin, as determined by CCK8 assay (n=3). **(E)** IC50 curves for intracellular MRSA inhibition by atractylenolide II and formononetin. The curves were generated by fitting a four-parameter logistic model to the data points, representing the mean of single independent experiments (n=3). Data are presented as the mean ± SD. Differences were analyzed applying ordinary one-way ANOVA followed by Dunnett´s multiple comparisons test (comparison with the Infected Model). *P < 0.05, **P < 0.01, ***P < 0.001.

CD40 is considered crucial for the macrophage-mediated inflammatory responses during MRSA persistent infection. A total of twenty prototype compounds from BFJD were tested for their ability to promote CD40 expression using flow cytometry. Based on concentrations determined by cell viability assays (**Supplementary Figure S5C**), the primary screening identified nine compounds that could promote the expression of CD40 in macrophages (**Figure 5A**). Furthermore, molecular docking was utilized to clarify the potential binding modes of these nine compounds with CD40 (PDB ID: 5DMI). As indicated in **Supplementary Table S5**, atractylenolide II and formononetin demonstrate high affinity for CD40, with binding energy values of −5.57 and −5.62 kcal/mol, respectively. Structural analysis revealed that atractylenolide II forms three hydrogen bonds with residues Glu150, Ala176, and Val152 (**Figure 5B**), whereas formononetin forms a single hydrogen bond with Pro44 (**Figure 5C**). Based on the concentrations indicated by the cell viability assays (**Figure 5D**), both atractylenolide II and formononetin demonstrated potent inhibitory effects on bacterial load. The IC50 values of these two active compounds were determined to be 9.716 μM and 46.79 μM, respectively (**Figure 5E**). In summary, these findings suggest that atractylenolide II and formononetin are key bioactive compounds in BFJD, contributing to its immunomodulatory effects and providing a foundation for further mechanistic exploration.

## 4 Discussion

Current research indicates that MRSA has developed resistance to multiple antibiotics, including aminoglycosides, macrolides, and fluoroquinolones. Consequently, alternative antibiotics such as vancomycin, linezolid, teicoplanin, and daptomycin are frequently employed in treatment (33). However, as chronic infections persist and antibiotic treatment fails, or adverse clinical events occur, MRSA continues to overcome various defense mechanisms and develop resistance to vancomycin and linezolid (34, 35). In our study, we discovered that BFJD can regulate the M1 polarization of macrophages and decrease the bacterial load in organs during persistent MRSA infection. Our research offers an alternative therapeutic strategy for the chronic infection of MRSA.

Macrophages, as a crucial component of the innate immune system, play a key role in the host’s defense against MRSA. Upon MRSA invasion, macrophages are activated and undergo M1 polarization. M1 type macrophages produce ROS, IL-1β, IFN-γ, and other pro-inflammatory cytokines to exert their phagocytic and antimicrobial effects (36). Mice deficient in macrophages and those with impaired CD11b expression exhibit increased mortality and a higher bacterial load in organs when infected with MRSA bloodstream infections (37, 38). Although macrophages can phagocytose and kill MRSA, a small fraction of the bacteria can interfere with macrophage recruitment and phagocytosis, thus evading the immune system (11). Additionally, MRSA can hide within macrophages, using them as an intracellular niche and spreading infection in a “Trojan horse” fashion (39). As a result, intracellular bacterial infection plays a significant role in the persistence and recurrence of chronic MRSA infections. Studies have shown a high correlation between macrophage polarization and MRSA persistent infection. MRSA promotes M2 polarization, inhibiting phagocytosis and inflammatory responses to maintain its intracellular survival (9, 40). Currently, immunomodulation focusing on macrophage repolarization holds promise for enhancing the treatment of MRSA persistent infection. This includes the use of nanocarrier technology to encapsulate IFN-γ, which promotes the reprogramming of macrophages from the M2 to the pro-inflammatory M1 phenotype (9), and the application of complement activators to reactivate the pro-inflammatory activity of local macrophages (41).

BFJD, a traditional Chinese medicine formula, has been clinically applied for the treatment of chronic pulmonary infections and has been utilized for over a decade in managing multidrug-resistant tuberculosis (17). The outcomes of MIC tests and bacterial load assessments in an acute and subacute infection animal model indicate that BFJD does not possess a direct antibacterial effect against MRSA. Nonetheless, during MRSA persistent infections, BFJD notably diminishes bacterial load and enhances macrophage recruitment and polarization within the lungs, suggesting that BFJD might exert its antibacterial effects by modulating the functional states of macrophage. Consequently, we employed an MRSA persistent infection cell model, which can mimic the persistent infection state *in vivo*, and the findings indicated that BFJD can promote M1 polarization and decrease intracellular bacterial load. Moreover, as an oxazolidinone antibiotic, linezolid inhibited MRSA growth with an MIC of 2 μg/ml; however, even at 6 μg/ml (3×MIC), linezolid was unable to reduce intracellular bacterial load, implying that MRSA exploits its intracellular niche to develop resistance against extracellular antibiotics (42, 43). Furthermore, flow cytometry identified a subset of macrophages expressing both CD80 and CD206 in the BFJD group. Previous studies have suggested that this subset represents a transitional state between M1 and M2 macrophages and has the ability to transition into M1 (44). These cells are crucial in maintaining the equilibrium between antibacterial immunity and immune tolerance (45, 46).

CD40, which is widely expressed on macrophages and other antigen-presenting cells, can drive pro-inflammatory responses and M1 polarization (47). Research has demonstrated that CD40 regulates host protective immunity by inducing the production of IL-12, NO, ROS, and other molecules (48). The absence of CD40 signaling weakens macrophage polarization towards the pro-inflammatory state (49) and even promotes host mortality under low-dose infection condition. In cancer research, CD40-targeted therapy is capable of re-educating M2, demonstrating its ability to regulate macrophage polarization (50). Additionally, CD40, as a member of the tumor necrosis factor receptor superfamily, activates the CD40-TRAF signaling cascade, promoting the MAPK pathway, inducing pro-inflammatory gene expression, and activating oxidative stress-related enzymes to directly enhance ROS production (51, 52), thereby contributing to pathogen clearance. Immunofluorescence and flow cytometry analyses indicate that BFJD augments intracellular ROS production, which aids in reducing the intracellular bacterial load. Nevertheless, we noticed inconsistencies between the total ROS and MitoSOX measurements. DCFH-DA assesses a broad spectrum of ROS, encompassing H_2_O_2_, ·OH, and ONOO^−^, whereas MitoSOX Red specifically targets mitochondrial superoxide with high precision. The observed increase in total cellular ROS (measured by DCFH-DA), along with a non-significant trend in mitochondrial O_2_·^−^ (detected by MitoSOX Red), suggests that BFJD may preferentially modulate cytosolic ROS generation over MitoSOX production. This could mitigate potential oxidative damage to cellular components from excessive MitoSOX levels (53). Subsequently, NAC intervention inhibits the effect of BFJD, suggesting that ROS is a key factor in the immunomodulatory mechanism of BFJD. Meanwhile, as a second messenger, ROS plays a crucial role in cellular signal transduction (54). The expression of the NF-κB reporter gene in infected cells was enhanced under BFJD intervention, but this effect was also suppressed by NAC. In summary, these findings suggest that BFJD could bolster the antibacterial effects of macrophages against intracellular MRSA through the CD40-ROS-NF-κB signaling pathway.

Exploring the interactions between active compounds in herbs and diseases is crucial for understanding the mechanisms of TCM formula against MRSA persistent infection. In our study, two active compounds—atractylenolide II and formononetin—were identified. These compounds exhibited significant inhibition of intracellular bacterial load and promoted CD40 expression on macrophages. Moreover, atractylenolide II and formononetin demonstrated good binding affinity with CD40 in molecular docking. Recent research indicates that atractylenolide II can inhibit M2 macrophage polarization (55) and promote ROS production (56). These results suggest that the synergistic interactions of multiple components and targets may underlie antimicrobial activity of BFJD against MRSA persistent infection.

## 5 Conclusion

**Figure 6:**
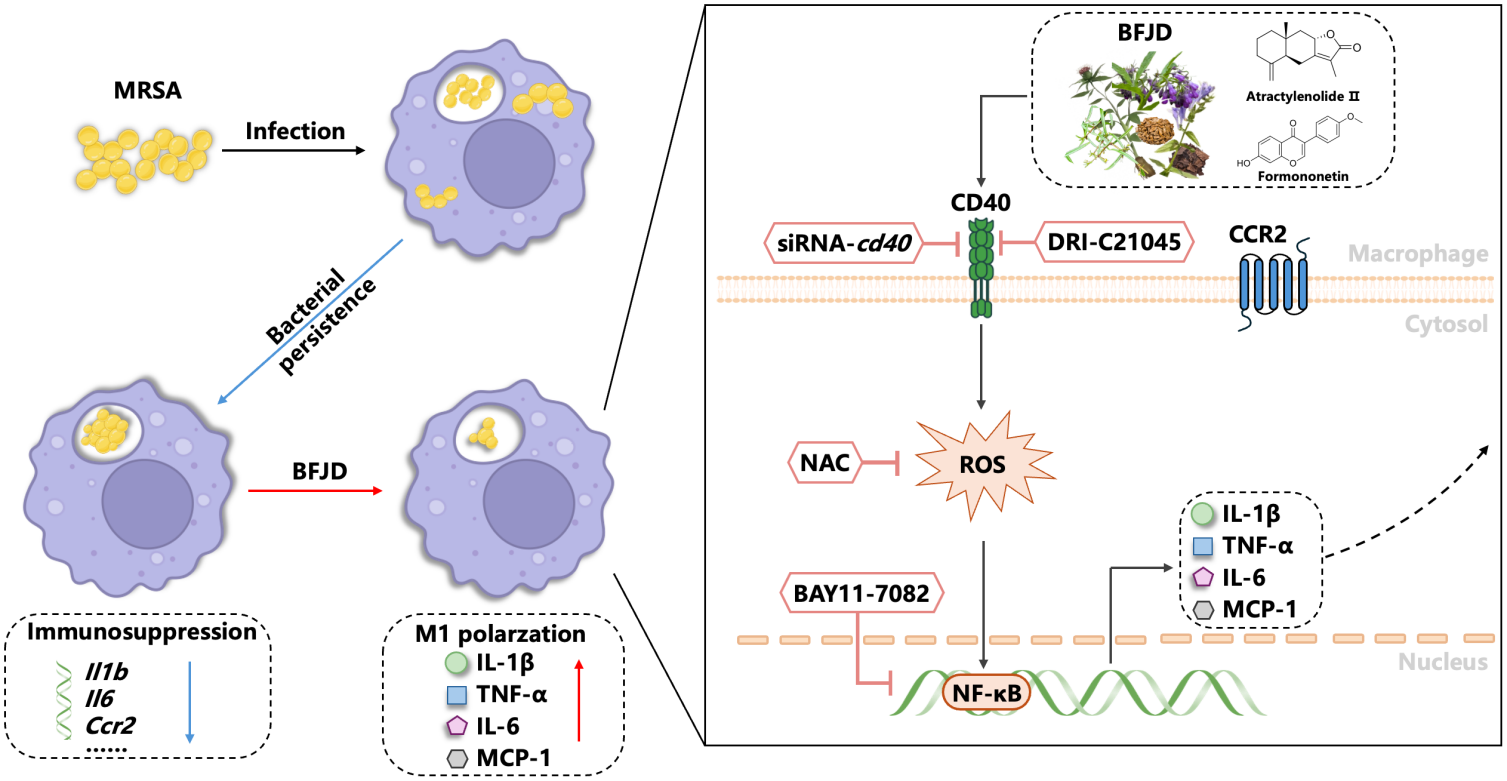
Model depicting how BFJD induces M1 polarization of macrophages via the CD40-ROS-NF-κB signaling pathway to combat persistent MRSA infection. Persistent MRSA infection employs immune evasion strategies by suppressing the expression of inflammatory genes in macrophages. BFJD stimulates the CD40-ROS-NF-κB signaling pathway, resulting in the production of ROS, IL-1β, IL-6, and TNF-α, thereby rekindling the macrophages’ antibacterial immune responses against persister cells. Through additional screening, we also identified two bioactive compounds, atractylenolide II and formononetin, which can activate the CD40 signaling pathway and similarly enhance the macrophages’ clearance of intracellular MRSA.

This study demonstrates that BFJD exerts its host-mediated antibacterial effects against MRSA persistent infection through the CD40-ROS-NF-κB signaling pathway. The identification of active compounds in BFJD that modulate immune functions and their potential antibacterial properties provides a new molecular basis for its clinical application (**Figure 6**). These findings will offer host-directed therapy and complementary alternative treatment strategies for multidrug-resistant bacterial infections or chronic bacterial infections.

## Data Availability

Original contributions presented in this study are included in the article/**Supplementary Material**. The sequencing dataset generated from this study has been deposited in the NCBI database under the GEO accession number GSE287681 (https://www.ncbi.nlm.nih.gov/geo/query/acc.cgi?acc=GSE287681). Further inquiries can be directed to the corresponding author.

## Supplementary material

Supplementary Figure 1 The antibacterial effect of Bufei Jiedu Formula (BFJD) in acute and subacute MRSA infection models. **(A)** Schematic illustration of a 4-day (acute) and 12-day (subacute) MRSA infection model. Mice were inoculated with 1×10^8^ CFU of MRSA in 0.2 ml of PBS via a lateral tail vein. In the acute infection model, BFJD intervention was administered immediately after infection, and the animals were euthanized for organ CFU counting after 4 days of intervention. In the subacute infection model, BFJD intervention was initiated at 4 days post infection (dpi), and the animals were euthanized for organ CFU counting on 12 dpi. **(B)** Bacterial burdens in the lungs, liver, and kidneys of mice (n=5).

Supplementary Figure 2 CCK8 assay of Bufei Jiedu Formula (BFJD). Cells were treated with different concentrations of BFJD (20, 200, 400, 800μg/mL) for 24 and 48h, and cell viability was determined using a cell counting kit-8 (n=5). Data are presented as the mean ± SD. Differences were analyzed applying ordinary one-way ANOVA followed by Dunnett´s multiple comparisons test (comparison with the untreated group). **P* < 0.05, ***P* < 0.01, ****P* < 0.001, *****P* < 0.0001.

Supplementary Figure 3 Analysis and verification of the transcriptome profile regulated by BFJD in intracellular MRSA persister infection *in vitro*. **(A-C)** Volcano plot displaying genes significantly upregulated (red) or downregulated (blue) from “M_24h vs Con”, “M_48h vs M_24h” and “BFJD vs M_48h”, respectively. **(D)** GO and KEGG enrichment analysis of the reversed DEGs of cluster I (391 DEGs). **(E)** Heatmap of the genes in the three most up-regulated KEGG pathways (cytokine-cytokine receptor interaction, NF-κB signaling pathway and TNF signaling pathway) (FC ≥ 1.5, adj p-value ≤ 0.05).

Supplementary Figure 4 Histogram analysis of Total ROS, MitoSOX, and CD40 expression in intracellular MRSA persister infection *in vitro*. **(A)** Overlapping histogram of ROS levels in the infected model, BFJD, and BFJD combined with the CD40 signaling inhibitor DRI-C21045. **(B)** Overlapping histogram of MitoSOX levels in the infected model, BFJD, and BFJD combined with the CD40 signaling inhibitor DRI-C21045. **(C)** Histogram of CD40 expression in the siRNA assay. **(D)** Histogram of ROS levels in the siRNA assay.

Supplementary Figure 5 Chemical profiling identification in the Bufei Jiedu Formula (BFJD) using UPLC-Q-TOF/MS system. **(A)** The chromatogram of the chemical base peak ion (BPI) from BFJD in the negative ion mode. **(B)** The BPI chromatogram of BFJD in the positive ion mode. **(C)** Cell viability of 20 active compounds at a single representative concentration (50 μM, n=3).
