# Supplementary figures and images for: Bufei Jiedu Formula enhances CD40 activation and macrophage polarization to eliminate intracellular MRSA persisters
Source: Front Immunol. 2025 Jul 17;16:1623182. doi: 10.3389/fimmu.2025.1623182 (PMC12310625; doi:10.3389/fimmu.2025.1623182)

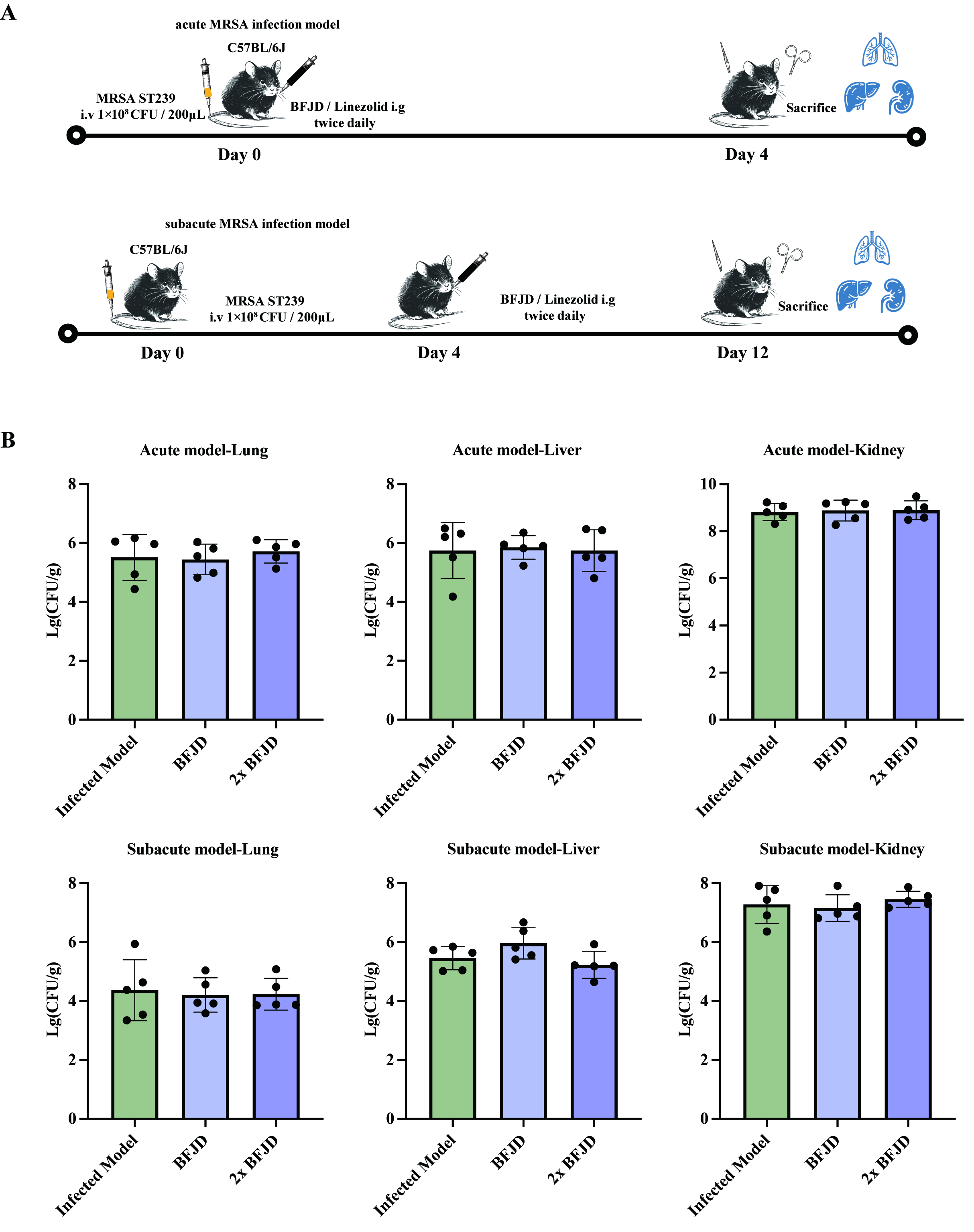

Supplement: Supplementary Figure 1 — The antibacterial effect of Bufei Jiedu Formula (BFJD) in acute and subacute MRSA infection models. (A) Schematic illustration of a 4-day (acute) and 12-day (subacute) MRSA infection model. Mice were inoculated with 1×10^8^ CFU of MRSA in 0.2 ml of PBS via a lateral tail vein. In the acute infection model, BFJD intervention was administered immediately after infection, and the animals were euthanized for organ CFU counting after 4 days of intervention. In the subacute infection model, BFJD intervention was initiated at 4 days post infection (dpi), and the animals were euthanized for organ CFU counting on 12 dpi. (B) Bacterial burdens in the lungs, liver, and kidneys of mice (n=5). [file Image1.tif]

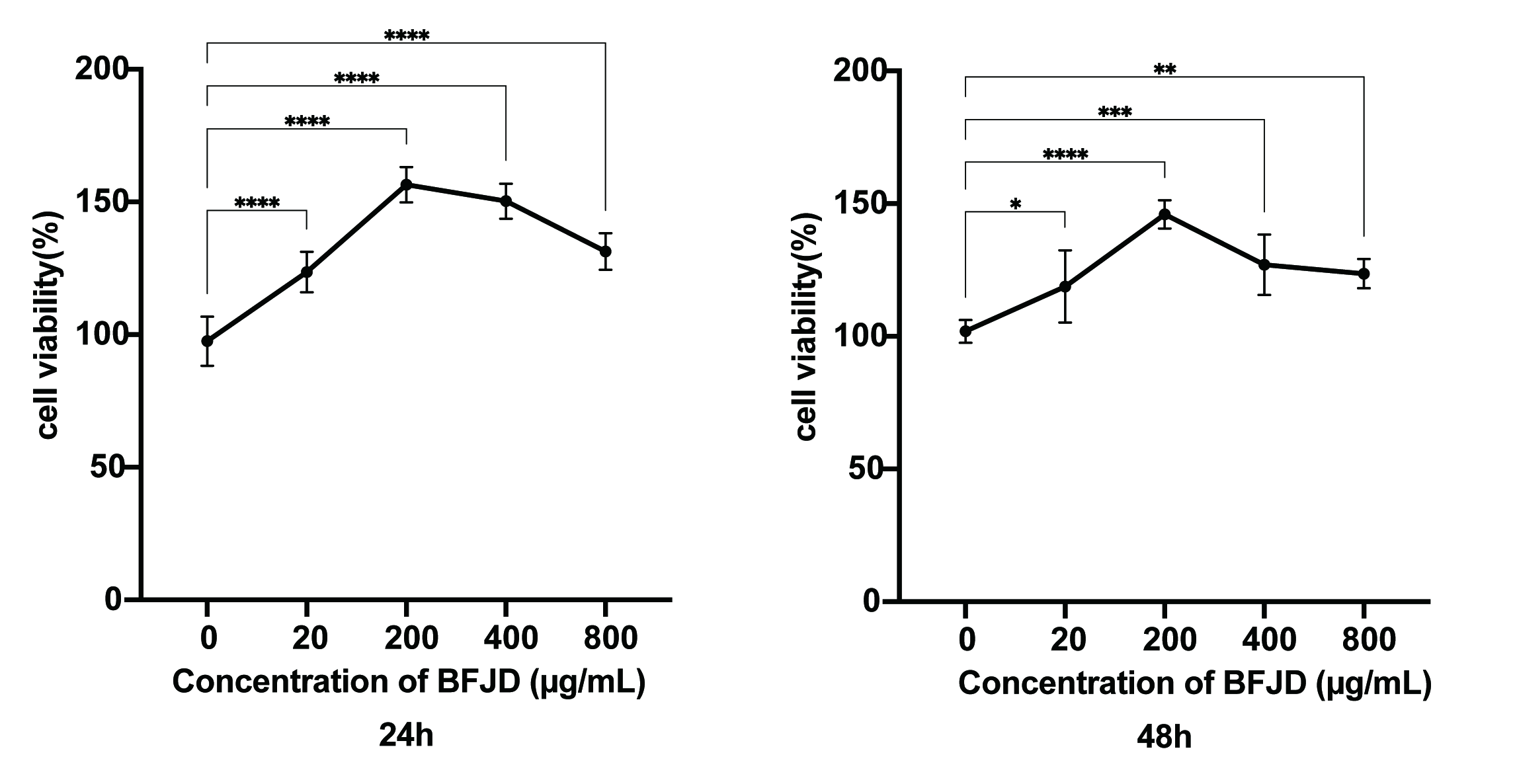

Supplement: Supplementary Figure 2 — CCK8 assay of Bufei Jiedu Formula (BFJD). Cells were treated with different concentrations of BFJD (20, 200, 400, 800μg/mL) for 24 and 48h, and cell viability was determined using a cell counting kit-8 (n=5). Data are presented as the mean ± SD. Differences were analyzed applying ordinary one-way ANOVA followed by Dunnett´s multiple comparisons test (comparison with the untreated group). *P < 0.05, **P < 0.01, ***P < 0.001, ****P < 0.0001. [file Image2.tif]

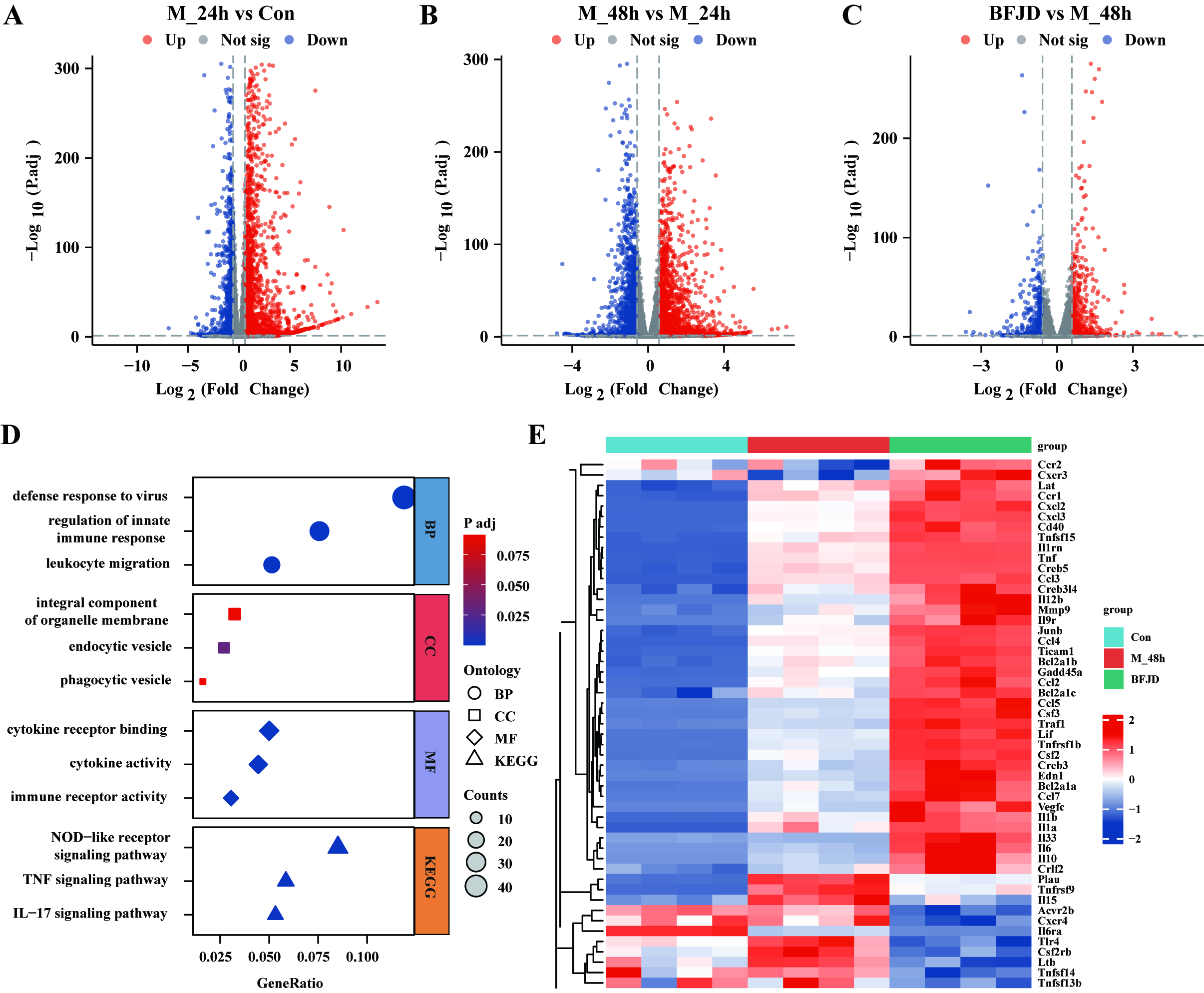

Supplement: Supplementary Figure 3 — Analysis and verification of the transcriptome profile regulated by BFJD in intracellular MRSA persister infection in vitro. (A-C) Volcano plot displaying genes significantly upregulated (red) or downregulated (blue) from “M_24h vs Con”, “M_48h vs M_24h” and “BFJD vs M_48h”, respectively. (D) GO and KEGG enrichment analysis of the reversed DEGs of cluster I (391 DEGs). (E) Heatmap of the genes in the three most up-regulated KEGG pathways (cytokine-cytokine receptor interaction, NF-κB signaling pathway and TNF signaling pathway) (FC ≥ 1.5, adj p-value ≤ 0.05). [file Image3.tif]

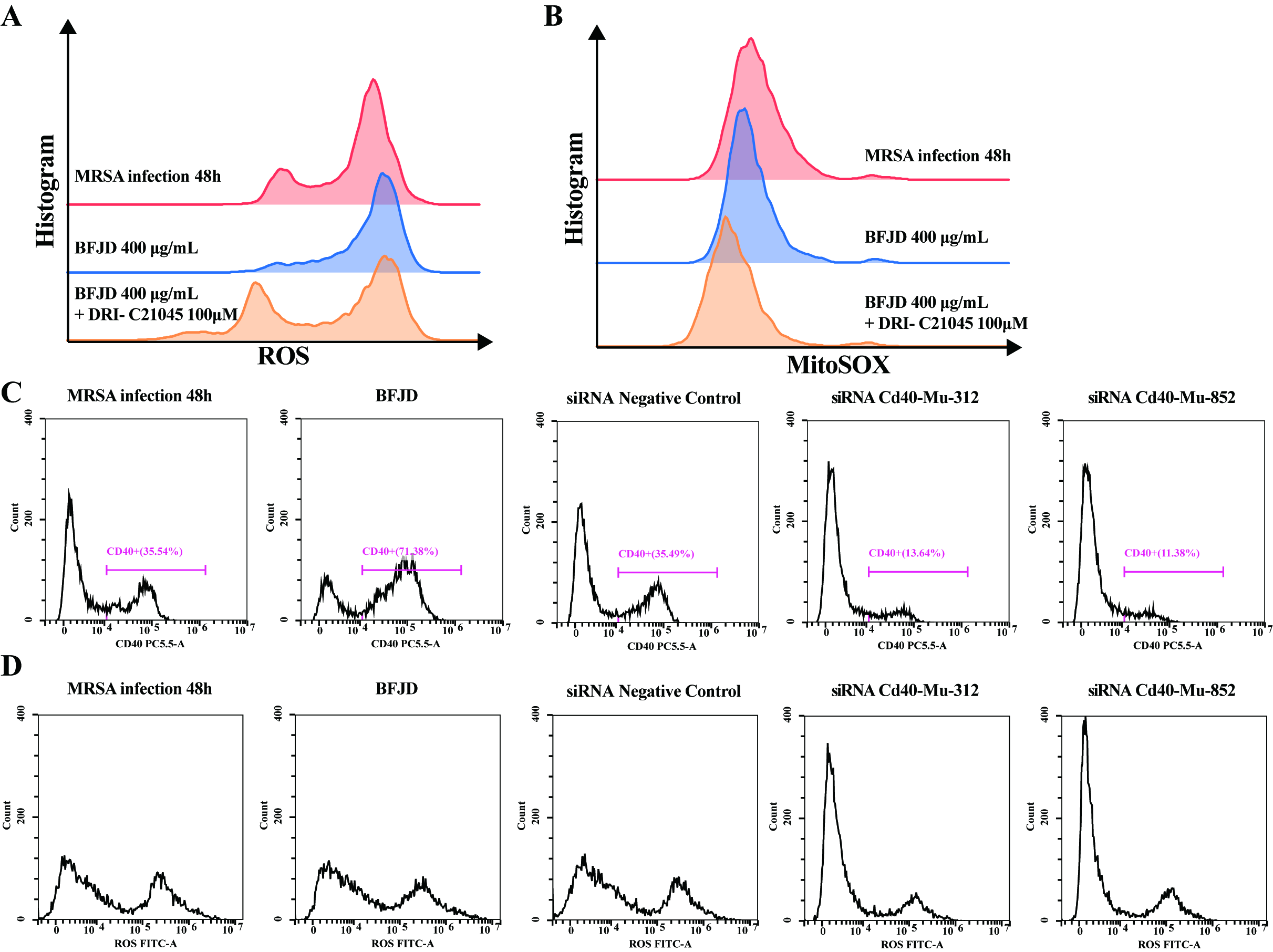

Supplement: Supplementary Figure 4 — Histogram analysis of Total ROS, MitoSOX, and CD40 expression in intracellular MRSA persister infection in vitro. (A) Overlapping histogram of ROS levels in the infected model, BFJD, and BFJD combined with the CD40 signaling inhibitor DRI-C21045. (B) Overlapping histogram of MitoSOX levels in the infected model, BFJD, and BFJD combined with the CD40 signaling inhibitor DRI-C21045. (C) Histogram of CD40 expression in the siRNA assay. (D) Histogram of ROS levels in the siRNA assay. [file Image4.tif]

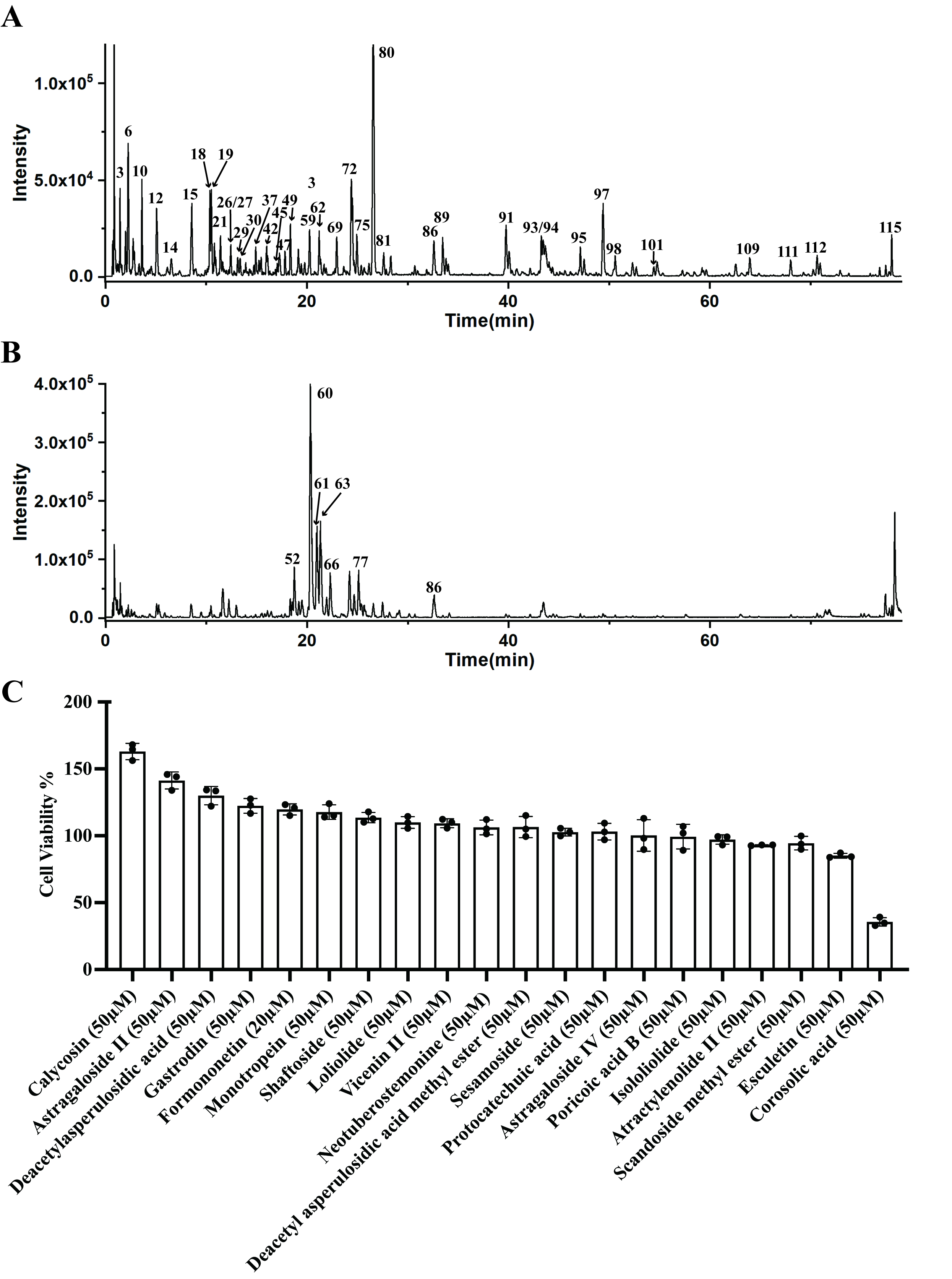

Supplement: Supplementary Figure 5 — Chemical profiling identification in the Bufei Jiedu Formula (BFJD) using UPLC-Q-TOF/MS system. (A) The chromatogram of the chemical base peak ion (BPI) from BFJD in the negative ion mode. (B) The BPI chromatogram of BFJD in the positive ion mode. (C) Cell viability of 20 active compounds at a single representative concentration (50 μM, n=3). [file Image5.tif]
