# Supplementary material for: Bufei Jiedu Formula enhances CD40 activation and macrophage polarization to eliminate intracellular MRSA persisters
Source: Front Immunol. 2025 Jul 17;16:1623182. doi: 10.3389/fimmu.2025.1623182 (PMC12310625; doi:10.3389/fimmu.2025.1623182)
Supplement: Supplementary file 8 [file Table3.docx]

Supplementary Table 3 Main chemical components of BFJD identified in the mouse serum and lung tissue

| Name | Formula | Retention time [min] | Ionization | Calc.  MW | m/z | serum | lung tissue | Chinese herbs |
| --- | --- | --- | --- | --- | --- | --- | --- | --- |
| Monotropein | C_16_H_22_O_11_ | 1.57 | [M-H]^-^ | 389.1084 | 389.108 | × | √ | BHSSC/LXC |
| Gastrodin | C_13_H_18_O_7_ | 2.13 | [M+Cl]^-^ | 321.0741 | 321.0746 | √ | × | BJ |
| Deacetylasperulosidic acid | C_16_H_22_O_11_ | 2.74 | [M-H]^-^ | 389.1084 | 389.1084 | × | √ | BHSSC/LXC |
| Protocatechuic acid | C_7_H_6_O_4_ | 4.65 | [M-H]^-^ | 153.0188 | 153.0185 | × | √ | BHSSC/JQM |
| Deacetyl asperulosidic acid methyl ester | C_17_H_24_O_11_ | 5.61 | [M+FA-H]^-^ | 449.1295 | 449.1298 | √ | √ | BHSSC/LXC |
| Scandoside methyl ester | C_17_H_24_O_11_ | 8.7 | [M+FA-H]^-^ | 449.1295 | 449.1293 | √ | √ | BHSSC |
| Sesamoside | C_17_H_24_O_12_ | 10.15 | [M-H]^-^ | 419.119 | 419.119 | √ | √ | XKC |
| Esculetin | C_9_H_6_O_4_ | 11.54 | [M-H]^-^ | 177.0188 | 177.0186 | √ | √ | ZHDD |
| Dactylorhin C | C_14_H_24_O_10_ | 12.29 | [M-H]^-^ | 351.1291 | 351.1297 | √ | √ | BJ |
| 3-Isobutylmalic acid | C_8_H_14_O_5_ | 12.88 | [M-H]^-^ | 189.0763 | 189.0766 | √ | √ | BJ |
| Croomine | C_18_H_27_NO_4_ | 15.01 | [M+H]^+^ | 322.2018 | 322.2016 | √ | √ | BB |
| Vicenin-II | C_27_H_30_O_15_ | 15.64 | [M-H]^-^ | 593.1506 | 593.15 | √ | √ | ZHDD |
| Shaftoside | C_26_H_28_O_14_ | 18.7 | [M-H]^-^ | 563.1401 | 563.1417 | × | √ | ZHDD |
| Loliolide | C_11_H_16_O_3_ | 19.78 | [M+H]^+^ | 197.1178 | 197.1172 | √ | √ | BB |
| Dactylorhin E | C_27_H_40_O_16_ | 20.01 | [M-H]^-^ | 619.2238 | 619.2234 | √ | √ | BJ |
| Isololiolide | C_11_H_16_O_3_ | 21.68 | [M+H]^+^ | 197.1178 | 197.1172 | √ | √ | BB |
| Neotuberostemonine | C_22_H_33_NO_4_ | 21.72 | [M+H]^+^ | 376.2488 | 376.2481 | × | √ | BB |
| Gymnoside Ⅰ | C_21_H_30_O_11_ | 24.15 | [M-H]^-^ | 457.171 | 457.1717 | √ | √ | BJ |
| Stemoninine | C_22_H_31_NO_5_ | 24.45 | [M+H]^+^ | 390.228 | 390.2272 | × | √ | BB |
| Calycosin | C_16_H_12_O_5_ | 32.28 | [M-H]^-^ | 283.0606 | 283.0625 | √ | × | HQ |
| Formononetin | C_16_H_12_O_4_ | 44.01 | [M-H]^-^ | 267.0657 | 267.0657 | × | √ | HQ |
| Astragaloside IV | C_41_H_68_O_14_ | 46.87 | [M+FA-H]^-^ | 829.4586 | 829.458 | √ | √ | HQ |
| Astragaloside II | C_43_H_70_O_15_ | 50.39 | [M+FA-H]^-^ | 871.4691 | 871.4731 | √ | × | HQ |
| Atractylenolide Ⅱ | C_15_H_20_O_2_ | 60.12 | [M+H]^+^ | 233.1542 | 233.1545 | √ | × | BZ |
| Corosolic acid | C_30_H_48_O_4_ | 66.95 | [M-H]^-^ | 471.3474 | 471.3464 | √ | × | LXC/XKC |
| Poricoic acid B | C_30_H_44_O_5_ | 68.25 | [M-H]^-^ | 483.311 | 483.3153 | × | √ | FL |
